# Decomposing the heterogeneity of depression at the person-, symptom-, and time-level: latent variable models versus multimode principal component analysis

**DOI:** 10.1186/s12874-015-0080-4

**Published:** 2015-10-15

**Authors:** Stijn de Vos, Klaas J. Wardenaar, Elisabeth H. Bos, Ernst C. Wit, Peter de Jonge

**Affiliations:** University of Groningen, University Medical Center Groningen, Interdisciplinary Center Psychopathology and Emotion regulation (ICPE), (internal mail CC-72), P.O. Box 30.001, 9700 RB Groningen, The Netherlands; University of Groningen, Johann Bernoulli Institute of Mathematics and Computer Science, Groningen, The Netherlands

**Keywords:** Data cube, Latent variable models, 3PCA, MPCA, Heterogeneity, Multimodal data, Depression

## Abstract

**Background:**

Heterogeneity of psychopathological concepts such as depression hampers progress in research and clinical practice. Latent Variable Models (LVMs) have been widely used to reduce this problem by identification of more homogeneous factors or subgroups. However, heterogeneity exists at multiple levels (persons, symptoms, time) and LVMs cannot capture all these levels and their interactions simultaneously, which leads to incomplete models. Our objective is to briefly review the most widely used LVMs in depression research, illustrating their use and incompatibility in real data, and to consider an alternative, statistical approach, namely multimode principal component analysis (MPCA).

**Methods:**

We applied LVMs to data from 147 patients, who filled out the Quick Inventory of Depressive Symptomatology (QIDS) at 9 time points. Compatibility of the results and suitability of the LVMs to capture the heterogeneity of the data were evaluated. Alternatively, MPCA was used to simultaneously decompose depression on the person-, symptom- and time-level and to investigate the interactions between these levels.

**Results:**

QIDS-data could be decomposed on the person-level (2 classes), symptom-level (2 factors) and time-level (2 trajectory-classes). However, these results could not be integrated into a single model. Instead, MPCA allowed for decomposition of the data at the person- (3 components), symptom- (2 components) and time-level (2 components) and for the investigation of these components’ interactions.

**Conclusions:**

Traditional LVMs have limited use when trying to define an integrated model of depression heterogeneity at the person, symptom and time level. More integrative statistical techniques such as MPCA can be used to address these relatively complex data patterns and could be used in future attempts to identify empirically-based subtypes/phenotypes of depression.

**Electronic supplementary material:**

The online version of this article (doi:10.1186/s12874-015-0080-4) contains supplementary material, which is available to authorized users.

## Background

Depression is a highly prevalent and burdensome disorder that is projected to become one of the largest contributors to the global burden of disease [1]. Although research has provided considerable insight into the mechanisms underlying depression, its phenomenology and (e.g. biological/psychological/environmental) origins have remained poorly understood. To improve this situation, several problems that have hampered research thus far should be overcome. A main problem for scientific research has been the lack of validity of currently used DSM/ICD-10 depression diagnoses [2, 3], which are not based on empirical evidence but on clinical consensus, leading to diagnoses with arbitrary boundaries and much overlap [4]. A second problem lies in the used syndrome approach to categorize patients, which allows for a great deal of heterogeneity among patients with the same diagnosis [3–6]. This problem is the focus of the current paper because, other than a total reconceptualization of the depression construct to improve its validity, decreasing heterogeneity can be done in currently available depression data using statistical techniques. Moreover, addressing this particular problem could already improve the specificity and interpretability of research results.

Heterogeneity is present in multiple aspects of depression: (1) the symptomatology of depression is heterogeneous, (2) the group of patients with a depression diagnosis is heterogeneous, and (3) the range of possible depressive course trajectories is heterogeneous. Consequently, it is unlikely that a few general etiological pathways could ever explain all possible patterns of depression onset, course and outcome. Although several neurobiological [7] and environmental [8, 9] factors have been shown to be generally involved in depression, observed effects have been small and hard to replicate.

**Fig. 1:**
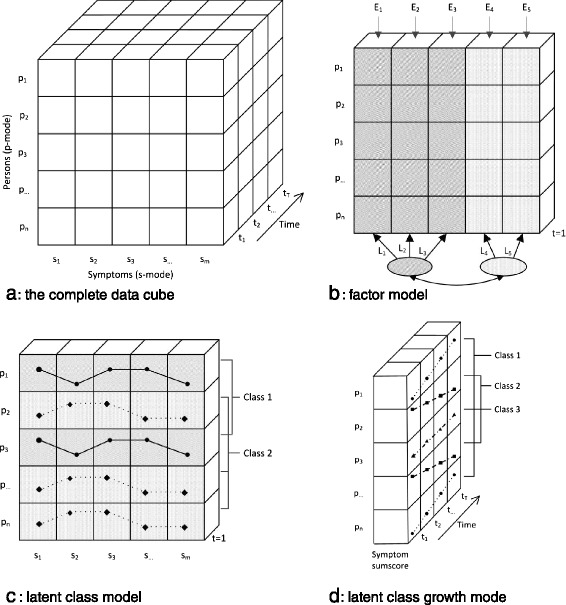
Slices of the data cube. Illustration of the data cube (**1a**), its different slices (**1b-d**) as well as associated techniques. p = person, s = symptom, t = time point

Data-driven statistical methods have been used to identify more homogeneous phenotypes that could enable investigation of more phenomenology-specific etiological pathways. So far, these approaches have been used to either identify symptom subdomains or patient subgroups based on common patterns of symptomatology. Statistically, this amounts to finding latent entities within sets of symptom data obtained from depressed patients [10]. A variety of latent variable models (LVMs) have been used for this and depending on the type of data and used LVM, different aspects of depression heterogeneity can be addressed. In an ideal scenario, symptom data are obtained from the same subjects at multiple time points, yielding a dataset with a three-modal structure: a person (p) mode, having *n* individuals, a symptom (s) mode, representing *m* psychometric variables and a time (t) mode, representing *T* measurements over time^1^. This data object can be represented mathematically as *X* = (*X*_*ijt*_), where *i* ranges from 1 to *n*, *j* from 1 to *m* and *t r*anges from 1 to *T*. A 3-mode data structure like this is often visualized as a ‘data cube’ (Fig. 1a) [11].

When addressing depression heterogeneity, LVMs are usually performed within a single ‘slice’ of the data cube (see Fig. 1b–d; adapted from [12]). In the cross-sectional slice, the data are represented by a *n × m* matrix *X* = (*X*_*ijk*_) where *k* is a fixed point in time (Fig. 1b and c). Two LVM approaches can be used to explore the latent structure of this slice: using (1) discrete or (2) continuous LV’s.

When using discrete LVs, latent subgroups of depression can be discerned based on their item responses (Fig. 1c). In the case of discrete observable variables a latent class model (LCM) is used. If observable variables are continuous, a latent profile model (LPM) is used [13]. A LCM/LPM typically includes one LV and assumes it to have a finite number of levels (i.e. classes), which are observed through a number of discrete variables (i.e. symptoms). To identify the optimal number of classes, various general (e.g. Bayesian Information Criterion [BIC], Akaike Information Criterion [AIC]) and specific [14] methods can be used. In depression research LCM and LPM approaches have been used to identify different subgroups of depressive patients (e.g. [10, 15–21]). For example, LCM identified classes of patients with ‘severe typical’ (decreased appetite/weight), ‘severe atypical’ (increased appetite/weight) and ‘moderate severe’ depression [18, 19]. In addition, LPM identified classes of patients with different profiles on the four depressive subdomains [21].

When using continuous LV’s, clustering of psychometric variables on underlying factors (Fig. 1b) can be investigated [20, 22]. Factor analysis is a well-known LVM using continuous latent variables (*factors*), which are measured through observable variables (e.g. symptoms, questionnaire items). With both exploratory factor analysis [23] and confirmatory factor analysis [24] we are interested in explaining the variability in the observable variables (e.g. symptoms, questionnaire data) by modelling common latent factors (e.g. depression severity). Factor analyses have been used widely to identify homogeneous subdomains of depressive symptomatology [20] and/or to investigate measurement scales’ structure/construct validity [22, 25].

In the longitudinal slice of the data cube (Fig. 1d), some variable *p*_*j*_ is fixed and responses from all individuals over all time points are considered. The data is represented by a *n × T* matrix *X* = (*X*_*ijk*_) for fixed *j*. Two aspects are of interest when accounting for heterogeneity in this data: (1) the modelling of individual *growth curves* and (2) evidence for the presence of subpopulations with respect to their growth. These models can be used to model the heterogeneity in the course trajectories of depression (e.g. [26]). Previous studies have mostly used discrete LVs (latent class growth models [LCGMs] and growth mixture models [GMMs]) and continuous outcomes to identify distinct depressive course groups (e.g. ‘quick remission’ vs. ‘chronic course’ [26–30]). With LCGMs, each person’s trajectory on a continuous outcome (e.g. depression severity) is assumed to belong to one of a number of classes. Each class has its own growth-curve characteristics (e.g. intercept and slope means). A GMM is similar to a LCGM but also models of within-class heterogeneity by estimating growth-parameter (co)variances [31]. This added flexibility often leads GMMs to be more parsimonious and to fit better to the data than LCGMs [26, 31].

It is important to note that the results of above described techniques in different data slices yield incompatible results in terms of the modes in which heterogeneity is explained and in which homogeneity is assumed [12]. Consequently, the results of traditional LVM studies can be very informative about the heterogeneity in particular modes of the data, but cannot be directly integrated into a single framework to explain all depression heterogeneity: i.e. factor analytic results about symptom-domains do not inform us about the best way to subgroup patients and vice versa. Because traditional LVMs fail to account for simultaneous variability in multiple modes of depression data, current models of depression heterogeneity are still incomplete and/or oversimplified.

**Fig. 2:**
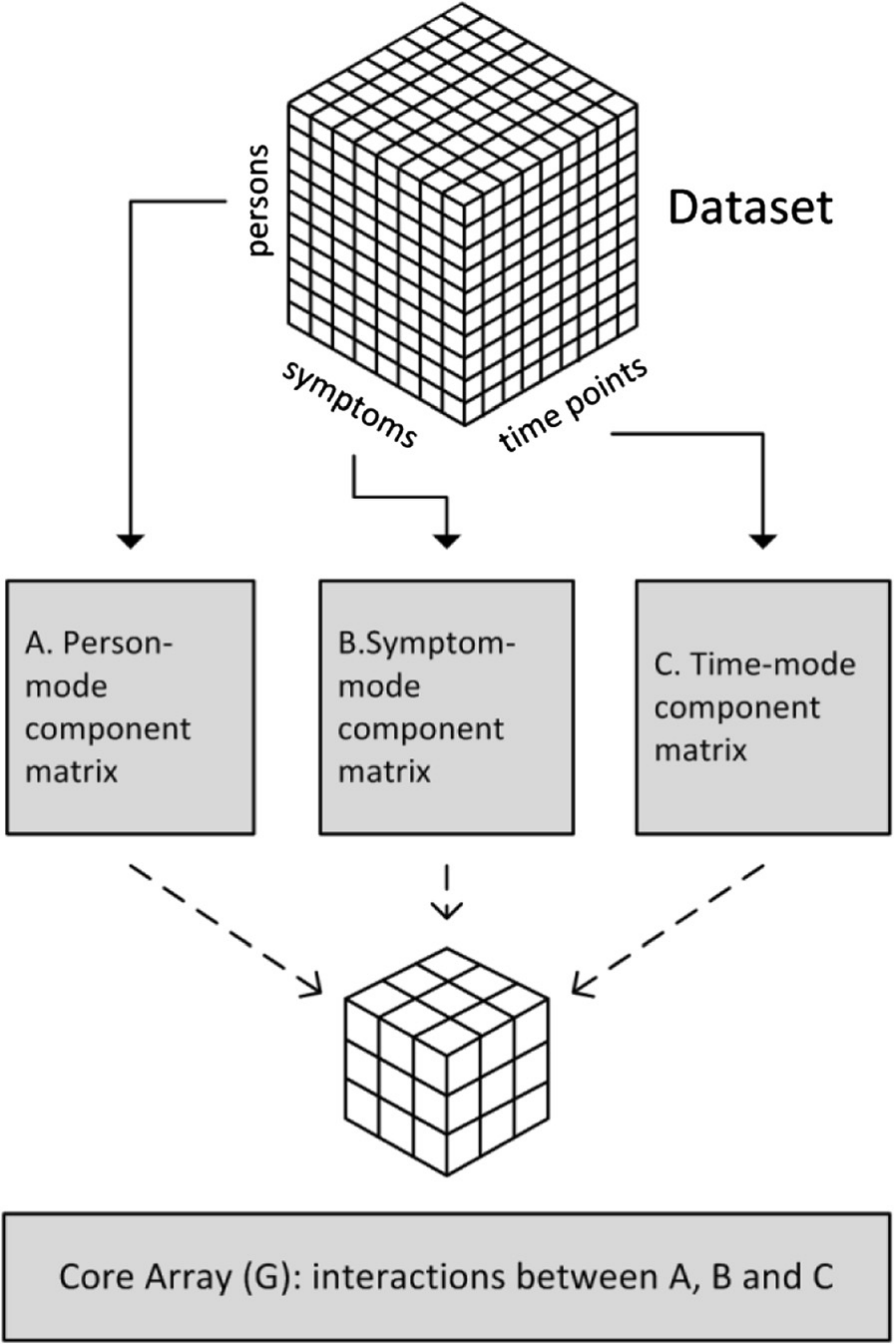
The elements of a 3-mode principle component model. Analysis of a 3-mode dataset yields a model consisting of a person-mode component matrix (**a**), a symptom-mode component matrix (**b**) and a time-mode component matrix (**c**). In addition, the interactions between the different modes’ components are described by the core-array (G)

A possible solution to this problem is to use statistical techniques that allow for simultaneous decomposition of depression on the person-, symptom- and time-level. This can be done with multiway principal component analysis (MPCA) [32]. MPCA is a higher-dimensional generalization of regular PCA that can be used to detect clustering tendencies in higher-dimensional datasets. Because we have three modes, we are interested in PCA models that work in three dimensions. There are various models that perform PCA in three dimensions, one of which is the Tucker3 model. This model is defined mathematically as

$$ {X}_{ijk}={\displaystyle \sum_{p=1}^P{\displaystyle \sum_{q=1}^Q{\displaystyle \sum_{r=1}^R{a}_{ip}{b}_{jq}{C}_{kr}{\mathit{\mathsf{g}}}_{pqr}+{e}_{ijk}}}} $$

where *X*_*ijk*_ is the entry in the three dimensional data set *X* at place (*i,j,k*) and *a*_*ip*_*b*_*jq*_*C*_*kr*_ are the elements of the component matrices A, B and C, respectively. The numbers ${\mathit{\mathsf{g}}}_{pqr}$ belong to the core array G and *e*_*ijk*_ represents the error of data entry *x*_*ijk*_. If the data set *X* contains data from *n* subjects, *m* symptoms and *T* time points and *P, R, Q* are the number of components in the A-, B- and C-mode respectively, then A has size *n* × *P*, B has size *m* × *Q* and C has size *T* × *R*. The core array is of size *P* × *Q* × *R*. See Fig. 2 for a graphical representation of the Tucker3 model. The component matrices A, B and C represent the loadings of the data entries onto their respective components. This can be compared to standard PCA, where, after picking a number of components, component scores are estimated. One difference between threeway PCA models and standard PCA is the core array G. This array contains numbers that represent how the components in the A-, B- and C-modes interact. It is this array that enables the integrated modeling of heterogeneity in three dimensional data. Without it, there would be no assumption of threeway interactions.

The interpretation of 3PCA component scores is similar as in the case of standard PCA. The symptom-mode components represent a characterization of groups of symptoms, the time-mode components illustrate groups of time trajectories and the person-mode components represent characterizations of groups of persons. The latter means that every person in the 3-mode dataset has a profile of scores on the person-mode components. In the context of depression research, these scores characterize the way in which in this person the symptom-components behave over time. Thus, the person-mode component scores provide person-specific characterizations, which can be interpreted in a similar way as, for instance, personality assessments where a person’s scoring profile on multiple traits gives a quantitative characterization of his personality.

Other threeway PCA models are obtained when constraints are imposed on the number of components per mode and/or the shape of the core array, yielding models such as the CANDECOMP/PARAFAC model. For an expansive overview of various multiway PCA models (including Tucker3), see [32].

This paper aims to illustrate and discuss the abovementioned problems and present the use of 3PCA as a possible solution. First, each of the LVMs is performed in an example depression dataset to illustrate their typical results. Second, the more integrative approach of 3PCA is demonstrated and its results are compared with the results of the traditional LVMs.

## Methods

To compare the results from all the above mentioned LVM techniques, they were run in a real-life, longitudinal depression dataset. EFA and LCA were conducted on the cross-sectional slice of this dataset and LCGMs and GMMs were applied to the longitudinal slice. 3PCA was performed on the whole dataset.

### Participants

The data came from 147 outpatients visiting a specialized day-care depression unit at the Department of Psychiatry at the University Medical Center Groningen (UMCG). The data collection was part of a 16 weeks long treatment program. The mean age was 42.9 (SD = 11.9), 50.8 % were female, and none were hospitalized. Patients were administered the Quick Inventory of Depressive Symptomatology (QIDS-SR; [33]) at baseline and, when possible, during every following week. The 147 patients with complete baseline data formed the cross-sectional data-slice (Fig. 1b and c) and were used for EFA and LCM. A subsample of 82 patients (mean age = 42.3 [sd = 12.1]; 51.2 % female) with at least nine complete weekly follow-up measurements was used for all longitudinal analyses (LCGM, GMM and 3PCA). For the EFA and LCM, baseline cross-sectional item-level QIDS-SR data were used. For the LCGM and GMM, aggregate QIDS-SR sum-scores at repeated time points were used. For 3PCA item-level QIDS data at each of the nine repeated time points were used.

The demographic data of the sample is summarized in Table 1.

**Table 1:** Sample descriptives

Data	Sample size	Mean age (s.d.)	% female
Cross sectional	147	42.9 (11.9)	50.3
Longitudinal	82 (9 time points)	42.3 (12.1)	51.2

*s.d.* standard deviation

### Participant secal

The data collection was part of a treatment program for patients at the Department of Psychiatry at the University Medical Center Groningen. At intake, patients are informed that the data collection is part of the general policy of the UMCG to monitor treatment outcome, that outcomes are made available only to their therapist and that the data will be used for research purposes, but only in anonymized form. If patients object to such use, their data are removed. The Dutch Central Committee on Research Involving Human Subjects (CCMO) approved the regulations and agreed with this policy. The study was conducted in accordance with the Declaration of Helsinki. The study protocol was approved by the medical ethical committee of the University Medical Center Groningen.

### Statistical guidelines

Reporting guidelines have been followed where applicable.

### Measurements

The QIDS consists of 16 items, coded on a 4-point Likert scale (0,1,2,3). Because the highest category (3) was endorsed very scarcely, this category was merged with category 2, resulting in a 3-point scale (0,1,2), which was more suitable for conducting LVMs with categorical data in Mplus.

Prior to analyses, hyposomnia items 1–3 were recoded into one item (item 1–3) using the highest score. Mutually exclusive items on increased and decreased appetite/weight were recoded into single appetite-change (item 6/7) and weight-change (item 8/9). For the longitudinal analyses, QIDS sum scores were computed for the nine consecutive time-points by adding up the item-scores at each time-point after recoding (item 1–3, item 6/7 and item 8/9).

### Statistical analyses

#### Latent variable models

All analyses (EFA, LCM, LCGM, GMM) were conducted with Mplus, version 6.12. [34]. Model estimation was done with Maximum Likelihood with robust standard errors (MLR). For each analysis, differently specified models (e.g. different class-numbers) were run. Model selection was performed by comparing the AIC and BIC, two common model selection criteria, between competing models.

EFA was conducted using the baseline QIDS-SR item-scores. During EFA, the optimal number of factors was determined by comparing the AIC and BIC between models with different numbers of factors. The default rotation method in Mplus (oblique Geomin) was used and the rotated factor loadings were inspected to interpret the content/meaning of each of the factors. Items were allocated to the factor on which they showed the highest factor loading. There exist other model selection methods such as the Scree-plot and parallel analysis. However, as the number of extracted factors was not the main point of interest in this study, these additional techniques were not considered. For the fitting of the LCMs, which was done using the baseline QIDS-SR item-scores, the profiles of item-endorsement probabilities in each class were plotted to interpret class differences.

LCGM and GMM fitting was done using the aggregate QIDS-SR sum scores at the 9 repeated time points. In both analyses, class-specific intercepts, slopes and quadratic terms were estimated. For LCGM, the variances of these terms were fixed at zero. For the GMMs random intercepts were estimated and slopes/quadratic terms were fixed.

### 3PCA

A 3PCA was performed in the longitudinal dataset (*n* = 82) using the item-level QIDS-data at each of the nine follow-up waves. The analyses were conducted with R package “ThreeWay” [35]. Data pre-processing (centering and normalization) and fit-percentage calculation was done following [36]. The pre-processing described there allows us to use the 3PCA analysis to investigate heterogeneity up-and-above the general mean trend in the dataset, which is shared by all patients ([35], p. 6). The ThreeWay R-package presents the user with modeling choices that have to be made during the analysis process, such as the number of components to be estimated per mode. There is also an option to calculate fit percentage for a variety of component combinations, after which the preferred number of components per mode can be selected. As is the case in standard PCA, model selection is non-trivial because a higher fit percentage can always be achieved by selecting more components. Taking into account fit percentage and the number of parameters in the model, we selected a model with, respectively 3, 2 and 2 components for the person-, symptom- and time-mode. The 3PCA model (the number of components per mode) was selected based on the percentage of explained variance.

The used R and Mplus scripts are available in Additional file 1 (Mplus scripts) and Additional file 2 (R scripts) to this paper.

## Results

### EFA

EFA results (Table 2) showed that based on the BIC, a 1-factor model would be deemed optimal; all QIDS items loaded high on one factor (Table 3), except for hypersomnia (item 4) and weight change (item 8/9) suggesting potential multidimensionality. Indeed, a decreasing AIC suggested that a second factor could be added. In the 2-factor model, the hypersomnia (item 4) and weight change items loaded on one ‘vegetative’ factor and all other items loaded on a second ‘mood/cognitive’ factor. The AIC favoured addition of a third factor, but this model had two factors with only one item loading on it. These results showed that symptomatology could be described by a 1-factor model, but that a 2-factor model was more informative in terms of differentiation between mood/cognitive and vegetative domains.

**Table 2:** Model assessment with maximum likelihood with robust standard errors (MLR)

Analysis type		Degrees of freedom	AIC	BIC
EFA (*n* = 147)	1-factor	36	3093	3201
	2-factor	47	3090	3230
	3-factor	57	3082	3252
LCM (*n* = 147)	1-class	24	3460	3532
	2-class	49	3161	3307
	3-class	74	3097	3318
	4-class	99	3071	3366
	5-class^a^	124	3052	3422
LCGM (*n* = 82)	1-class	12	4670	4699
	2-class	16	4363	4401
	3-class	20	4267	4315
	4-class	24	4220	4278
	5-class^a^	28	4173	4240
GMM (*n* = 82)	1-class	15	4188	4224
	2-class	21	4163	4213
	3-class	27^b^	4146	4210
	4-class	33^b^	4158	4237

*FA* factor analysis, *LCM* latent class models, *LCGM* latent class growth models, *GMM* growth mixture models

^a^smallest class contains < 10 subjects

^b^smallest class contains < 5 subjects

**Table 3:** Rotated factor loadings of the 1- and 2-factor model

QIDS item number	Item label	1-factor model	2-factor model	
			‘Mood/Cognitive’	‘Vegetative’
1–3	Hyposomnia	**0.35**	**0.37**	−0.23
4	Hypersomnia	0.14	0.00	**0.85**
5	Feeling sad	**0.89**	**0.88**	0.00
6/7	Changed appetite	**0.40**	**0.36**	0.25
8/9	Changed weight	0.12	0.06	**0.36**
10	Concentration	**0.85**	**0.88**	−0.13
11	View of myself	**0.69**	**0.67**	0.06
12	Thoughts of death/suicide	**0.66**	**0.61**	0.32
13	General interest	**0.78**	**0.79**	−0.07
14	Energy level	**0.69**	**0.69**	0.01
15	Psychomotor retardation	**0.56**	**0.56**	0.02
16	Psychomotor agitation	**0.39**	**0.41**	−0.08

*QIDS* quick inventory of depressive symptomatology

Loadings after oblique Geomin rotation

Factor loadings were boldfaced to indicate to which factor the corresponding item belongs to

### LCM

**Fig. 3:**
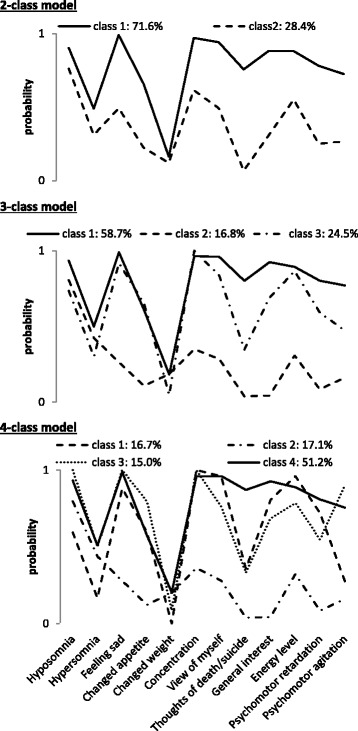
LCA item probabilities. Item probabilities for 2-, 3-, and 4-class latent class models in a sample of 147 help-seeking patients. The y-axis denotes the probability of endorsing a non-zero response

LCM results are shown in Table 2. The 2-class model has the lowest BIC, whereas the AIC continued to decrease with each class addition. The 2-class model (Fig. 3) indicated that heterogeneity among persons could be explained by the existence of a low and a high severity class. Motivated by the AIC values, a 3-class model was also investigated, which showed a low-severity class (16.8 %), but further distinguished two more qualitatively different high-severity classes (see Fig. 3): one class (58.7 %) showed higher probabilities of endorsing suicidality, loss of interest, and psychomotor problems than the other high-severity class (24.5 %). The 4-class model showed additional qualitative differentiation between high-severity classes, with one class showing a relatively higher probability of suicidal ideation (51.2 %) and another of relatively higher psychomotor agitation (15.0 %; see Fig. 3).

### LCGM and GMM

**Fig. 4:**
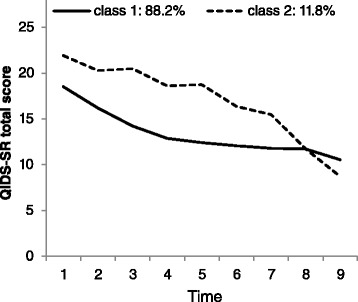
GMM trajectories. Class-specific trajectories of observed mean Quick Inventory of Depressive Symptoms (QIDS) scores for the best-fitting (GMM) in a sample of 82 help-seeking patients

GMM models fit better to the data than LCGM models as shown by lower AIC and BIC levels. In the GMM the AIC and BIC decreased until addition of a fourth class. However, the best fitting 3-class model had one small class (*n* = 3) that could not be reliably interpreted. Therefore, the 2-class model was selected for further inspection. The observed trajectories are shown in Fig. 4: one class (88.2 %) was characterized by a relatively quick decrease in symptomatology compared to the other class, which showed a slower decrease in severity.

### 3PCA

The results of the 3PCA analyses are shown in Tables 4, 5 and 6. Because it showed comparatively good fit to the data (explained variance = 80.3 %), a 3PCA model with three components in the person-mode, two in the symptom-mode and two in the time-mode was selected (3,2,2). The scores on the symptom components (Table 4) showed that the symptom mode could be decomposed into a somatic/affective and cognitive/appetitive component. The scores on the time components (Table 5) indicated that the initial 5 time points scored high on one component and time points 6 through 9 scored high on the a second time component. Considering that these scores represent variability up and above the general downward trend in the data, the first component was interpreted to represent a ‘persisting’ time component while the second component represented an ‘improving’ time component.

**Table 4:** 3PCA symptom component scores

QIDS item number	Item	1: somatic/affective	2: cognitive/appetitive
1–3	Hyposomnia	**0.36**	−0.19
4	Hypersomnia	−0.15	0.21
5	Feeling sad	**0.38**	0.12
6/7	Changed appetite	−0.01	**0.63**
8/9	Changed weight	0.03	**0.42**
10	Concentration	**0.39**	0.00
11	View of myself	0.16	**0.28**
12	Thoughts of death/suicide	0.13	**0.36**
13	General interest	**0.43**	−0.07
14	Energy level	**0.30**	0.14
15	Psychomotor retardation	0.25	0.19
16	Psychomotor agitation	**0.41**	−0.25

*3PCA* three-mode principle component analysis, *QIDS* quick inventory of depressive symptomatology

Component scores were boldfaced to indicate to which component the corresponding item belongs to

**Table 5:** 3PCA time component scores

Time points	1: persisting phase	2: improving phase
1	−0.19	**0.48**
2	−0.05	**0.50**
3	0.08	**0.45**
4	0.11	**0.40**
5	0.18	**0.35**
6	**0.35**	0.16
7	**0.49**	−0.02
8	**0.55**	−0.10
9	**0.50**	−0.04

*3PCA* three-mode principle component analysis

Component scores were boldfaced to indicate to which component the corresponding item belongs to.

**Table 6:** 3PCA core component scores

Person components		Symptom component	
	Time component	1: somatic/affective	2: cognitive/appetitive
1: early recovery	1: improving phase	24.55	10.38
	2: persisting phase	5.80	−0.89
2: persistent somatic/affective	1: improving phase	6.05	−18.89
	2: persisting phase	2.75	−15.89
3: increasing symptoms	1: improving phase	16.61	4.73
	2: persisting phase	28.60	10.50

*3PCA* three-mode principle component analysis

The characteristics of the person-mode components could be deduced from the core-array (Table 6), which quantifies the interactions between the symptom- and time-mode components per person-mode. The first person-mode was characterized by comparatively high somatic/affective and cognitive/appetitive symptom-component scores in the first, ‘improving’ time-phase and much lower symptom-component scores in the ‘persisting’ time-phase. As such, persons with high scores on the first person-mode component were characterised by ‘*overall quick recovery*’. The second person-mode component was characterized by much higher somatic affective than ‘cognitive/appetitive’ symptom-component scores in the ‘improving’ time-phase and this situation remained the same for the ‘persisting’ time-phase. Therefore, scores on this person-mode components were associated with ‘*persistent somatic/affective symptomatology*’. The third person-mode component was characterized by slightly different ‘somatic/affective’ and ‘cognitive/appetitive’ scores in the ‘improving’ time-phase, which both increased in the ‘persisting’ time-phase. Scores on this component were thus associated with ‘*overall increasing symptomatology’*.

### LVMs versus 3PCA

Some of the traditional LVM results showed parallels with some aspects of the 3PCA results. The 3-PCA symptom-mode components showed quite some overlap with the results of the EFA: items 1–3, 5, 10 and 13–16 were grouped similarly in both models, although the component scores of items 11 and 12 (appetite change and weight change) in the 3PCA model made the interpretation different from that of the EFA model.

The time-mode components cannot be directly compared to the growth model analysis because the former is interpreted as two components of the mode time, whereas the latter is interpreted in terms of depression growth-curves over time. In as similar vein, the person-mode components cannot be compared to the LCM results because the former characterize persons in terms of symptom-component scores over time, whereas the latter characterizes persons based only on their symptom-score profiles. As such, the core-array of the 3PCA adds an integrative aspect to the analyses that is absent from the traditional techniques and allows for the description of person-heterogeneity in terms of symptom-scores across time.

## Discussion

Although LVMs can be used to address the heterogeneity of depression in particular slices of a three-modal data set, LVMs used so far in depression research are unsuited to form a complete picture of depression heterogeneity, integrating all sources of heterogeneity at the same time. As such, traditional LVMs are suboptimal to find a solution to the heterogeneity problem. Aside from the incompatibility of the results obtained from different LVMs and different data-slices, the assumptions of local independence of the symptoms and the independence of the different data-slices are unrealistic.

An alternative for traditional LVMs was presented in a 3PCA approach, which describes heterogeneity in three-modal data in a more integrated manner by simultaneously decomposing each mode into several components and considering the interactions between the different sources of heterogeneity. Although decomposing a single mode into a number of components by itself is not an improvement over more traditional LVMs, 3PCA has the added value that it allows for a quantification of the relationship between the various modes’ components. Since heterogeneity is difficult to describe by looking at any mode on its own, the core array offers interesting new insight into the heterogeneity of depression. When applied in research, this means that, rather than just showing how depressive symptomatology can be decomposed into symptom domains or showing how a sample can be decomposed into subtypes based on symptom profiles, 3PCA provides a more integrated insight into how persons differ in terms of their course-trajectories on different symptom-domains. Although 3PCA results can sometimes be hard to interpret, the presented results clearly illustrate its use to gain more insight into the nature and extent of heterogeneity among depressive patients in a given sample, providing an integrated picture of how patients differ from each other in terms of their course-trajectories on different symptom domains.

### Limitations

The results of the current study should be interpreted taking into account some study limitations. Firstly, the sample size (*N* = 82) for the longitudinal analyses is arguably small, while the number of time points is limited (T = 9). This is a consequence of the number of participants having nine or more measurements; only 82 of them had nine measurements. Consequently, model results might be influenced by the difference in sample sizes. Second, there exist numerous ways to perform traditional LV analyses. For example, it is conceivable that a different rotation method would have resulted in somewhat different factor loadings, which would also affect the comparability with the 3PCA symptom-mode components. Third, the factor analysis and latent class analysis was performed on data coming from the 147 patients, while the longitudinal analyses were performed on data from a subset of those patients (*n* = 82). The latter was due to the fact that in its current form, 3PCA cannot handle missing data.

Future studies should investigate 3PCA in larger samples and incorporate a larger number of symptoms and follow-up measurements. Moreover, the scientific and/or clinical relevance of 3PCA components should be evaluated.

## Conclusions

Investigation of heterogeneity by using LVMs cannot provide insight into all sources of heterogeneity simultaneously. The complexity of the problem requires the development of techniques that investigate patient subdivision based on their symptomatology and course. To this end, MPCA might prove to be a valuable alternative.

## Availability of supporting data

The data used in this study is available from the corresponding author upon request.

## Additional files

Additional file 1:
**A pdf file containing the Mplus scripts for the presented latent variable models.** (PDF 30 kb) Additional file 2:
**A pdf file containing the R script for the presented multiway principle component analysis.** (PDF 25 kb)

## Abbreviations

DSM: Diagnostic and statistical manual
ICD-10: International classification of disease 10
LVM: Latent variable model
LCM: Latent class model
LPM: Latent profile model
LCGM: Latent class growth model
GMM: Growth mixture model
EFA: Exploratory factor analysis
CFA: Confirmatory factor analysis
MPCA: Multiway principal component analysis
3PCA: Three-way principal component analysis

## Footnotes

1 What we here refer to as a mode is known as a dimension in mathematics. This is in contrast with medical jargon, where a mode (e.g. symptom mode) contains various dimensions (e.g. positive affect, negative affect).
