# Supplementary material for: Decomposing the heterogeneity of depression at the person-, symptom-, and time-level: latent variable models versus multimode principal component analysis
Source: BMC Med Res Methodol. 2015 Oct 15;15:88. doi: 10.1186/s12874-015-0080-4 (PMC4608190; doi:10.1186/s12874-015-0080-4)
Supplement: Additional file 1: — A pdf file containing the Mplus scripts for the presented latent variable models. (PDF 30 kb) [file 12874_2015_80_MOESM1_ESM.pdf]

Additional File 1

Supplement to: de Vos et al., Decomposing the Heterogeneity of Depression at the Person-, Symptom-, and Time-level: Latent Variable Models versus Multimode Principal Component Analysis

Annotated Mplus scripts for different latent variable models.

1. Exploratory Factor Analysis:

```
data: file is "data.dat";

listwise is on; ! listwise deletion of cases with missing data

Variable:
names are ! assign variable names to columns in data.dat
id qids1 qids4 qids5
qids6 qids8 qids10 qids11 qids12
qids13 qids14 qids15 qids16;

usevariables are  ! select variables to be included in the analysis
qids1 qids4 qids5
qids6 qids8 qids10 qids11 qids12
qids13 qids14 qids15 qids16;

categorical are   ! identify categorical variables
qids1 qids4 qids5
qids6 qids8 qids10 qids11 qids12
qids13 qids14 qids15 qids16;

missing are all (-99);

analysis:  type = efa 1 5; ! run EFA for 1- to 5-factor models
           rotation = geomin; ! use geomin rotation (default)
           estimator = mlr; ! use MLR estimator
           algorithm = integration;          !use numerical integration
           integration= standard (5);         ! number of integration points
plot:      type = plot2; !output Scree-plot
```

2. Latent Class Analysis

```
data: file is "data.dat";

listwise is on;

Variable:
names are id qids1 qids4 qids5
qids6 qids8 qids10 qids11 qids12
qids13 qids14 qids15 qids16;

usevariables are qids1 qids4 qids5
qids6 qids8 qids10 qids11 qids12
qids13 qids14 qids15 qids16;

categorical are qids1 qids4 qids5
qids6 qids8 qids10 qids11 qids12
qids13 qids14 qids15 qids16;

    classes = c (2);             ! number of classes to estimate

analysis:

    type=mixture ;
    starts=100 10;          ! set number of random starts
    process=8 (STARTS);     ! divide calculations across processors
    lrtstarts= 0 0 100 10; !Set number of starts for BLRT
    lrtbootstrap=100;       !Set number of bootstraps for BLRT

output: tech14; ! calculate the bootstrapped LRT
plot: ! generate plots for the LCA models
series = qids1 - qids16 (*);
type=plot3;
```

3. Latent Class Growth Analysis

```
DATA: file="data.dat";

listwise is on;

Variable:

names are id ss1 ss2
ss3 ss4 ss5 ss6 ss7
ss8 ss9;

usevariables are ss1
ss2 ss3 ss4 ss5 ss6
ss7 ss8 ss9;

classes=c(2); ! estimate a 2-class model

Analysis:
type=mixture;
process= 8 (starts); starts=250 50;
process=8 (STARTS);
estimator=MLR;
lrtstarts= 0 0 100 20;
lrtbootstrap=100;

Model:
%overall% ! define the overall growth model
i1 s1 q1 | ss1@1 ss2@2 ss3@3 ss4@4 ss5@5
ss6@6 ss7@7 ss8@8 ss9@9;

! define class-specific growth model: parameter variances
! fixed to zero, parameter means freely estimated.
%c#1%
[i1 s1 q1];
i1-q1@0;

%c#2%
[i1 s1 q1];
i1-q1@0;

Plot: ! generate plot of observed and estimated growth curves
series = ss1 - ss9 (*) ;
TYPE=PLOT3;

output:
TECH14
```

4. Growth Mixture Modeling

```
data: file="data.dat";

listwise is on;

Variable:

names are id ss1 ss2
ss3 ss4 ss5 ss6 ss7
ss8 ss9;

usevariables are ss1
ss2 ss3 ss4 ss5 ss6
ss7 ss8 ss9;

Missing = all (-99);

classes=c(2); ! estimate a 2-class model

Analysis:
type=mixture missing;
process= 8 (starts);
starts=1000 100;
```

```
estimator=MLR;
lrtstarts= 0 0 250 25;
lrtbootstrap=100;

Model:
%overall%
i1 s1 q1 | ss1@1 ss2@2 ss3@3 ss4@4 ss5@5
ss6@6 ss7@7 ss8@8 ss9@9;

! define class-specific growth model: parameter variances and
! means both freely estimated.

%c#1%
[i1 s1 q1];
i1-q1*;

%c#2%
[i1 s1 q1];
i1-q1*;

Plot:
series = ss1 - ss9 (*) ;
TYPE=PLOT3;

output:
TECH14
```
