# Supplementary material for: Decomposing the heterogeneity of depression at the person-, symptom-, and time-level: latent variable models versus multimode principal component analysis
Source: BMC Med Res Methodol. 2015 Oct 15;15:88. doi: 10.1186/s12874-015-0080-4 (PMC4608190; doi:10.1186/s12874-015-0080-4)
Supplement: Additional file 2: — A pdf file containing the R script for the presented multiway principle component analysis. (PDF 25 kb) [file 12874_2015_80_MOESM2_ESM.pdf]

Additional File 2

Supplement to: de Vos et al., Decomposing the Heterogeneity of Depression at the Person-, Symptom-, and Time-level: Latent Variable Models versus Multimode Principal Component Analysis

R-script for 3-way PCA.

```
# All model fitting was done with the function T3(), which implements a 3PCA model.
# The input data needs to be an
# "Array of order n x m x p or matrix or data.frame of order (n x mp) containing the
matricized array (frontal slices)".
# For more info, see "?T3" or

# P. Giordani, H.A.L. Kiers, M.A. Del Ferraro (2014).
# Three-way component analysis using the R package ThreeWay. Journal of Statistical
Software 57(7):1-23.
# http://www.jstatsoft.org/v57/i07/.

# To reshape the data from a long to a wide format, one can use the reshape2
library.
# Assuming the data is in an object called df:

if(!("ThreeWay" %in% installed.packages()) {
  install.packages("ThreeWay")
}

library(ThreeWay)

df.out <- T3(df)
```

Running T3() will start an interactive menu guiding the user in making various modelling choices. Note that the fit percentage reported in the manuscript was not obtained through this function, but by calculating it manually according to Monden et al., see reference #36 in the article.
